# Supplementary material for: Current Epidemiology of the General Anesthesia Practice for Cesarean Delivery Using a Nationwide Claims Database in Japan: A Descriptive Study
Source: J Clin Med. 2022 Aug 17;11(16):4808. doi: 10.3390/jcm11164808 (PMC9409718; doi:10.3390/jcm11164808)
Supplement: Supplementary file 1 [file jcm-11-04808-s001.zip › jcm-1811187-supplementary/Table S1.pdf]

Supplemental Table S1. List of all anesthesia-related codes.

Anesthesia information coded according to Japanese claims classification (anesthesia category: L)		
Code	Description	Basic payment *
L008	General anesthesia defined as follows: General anesthesia used in a closed-circuit system maintained by mask or mechanical ventilation. (1) General anesthesia in a closed or semi-closed system administered using a gas anesthesia machine for 20 minutes or more (2) General anesthesia agent administered intravenously and combined with oxygen/nitrous oxide mixture by mask or endotracheal intubation for 20 minutes or more	6,000 units
L004	Spinal anesthesia	850 units
L002	Epidural anesthesia	1,500 units (cervical/thoracic)<br>800 units (lumbar)<br>340 units (sacral)
L003	Continuous infusion of local anesthetic after epidural anesthesia (daily, excluding the day of anesthesia)	80 units per day (not including the day of anesthesia)
Drug prescription information coded according to the World Health Organization Anatomical Therapeutic Chemical (WHO-ATC) classification		
Code	Description	
M03	Peripherally, centrally, and directly acting muscle relaxants	

* One unit is claimed 10 Japanese Yen.
