# Supplementary material for: Current Epidemiology of the General Anesthesia Practice for Cesarean Delivery Using a Nationwide Claims Database in Japan: A Descriptive Study
Source: J Clin Med. 2022 Aug 17;11(16):4808. doi: 10.3390/jcm11164808 (PMC9409718; doi:10.3390/jcm11164808)
Supplement: Supplementary file 1 [file jcm-11-04808-s001.zip › jcm-1811187-supplementary/Table S2.pdf]

Supplemental Table S2. List of all ICD-10 code definitions of clinical indications for general anesthesia in the study

Clinical indication of general anesthesia for cesarian delivery	
Clinical indication	ICD-10 code
Obstetrical, maternal, fetal emergency	
Amniotic fluid embolism	O881
Chorioamnionitis	O411
Fetal distress (including abnormal fetal heart rate or rhythm)	O68
Obstetric hemorrhage	O67
Placental abruption	O45
Placenta accreta	O720
Placental dysfunction	O365
Umbilical cord prolapse	O69
Uterine rupture	O710
	O711

Based on the previous study [1]

[1]. Guglielminotti, J.; Landau, R.; Li, G. Adverse events and factors associated with potentially avoidable use of general anesthesia in cesarean deliveries. *Anesthesiology* **2019**, 130, 912–922. doi: 10.1097/ALN.0000000000002629.
