# Supplementary material for: Current Epidemiology of the General Anesthesia Practice for Cesarean Delivery Using a Nationwide Claims Database in Japan: A Descriptive Study
Source: J Clin Med. 2022 Aug 17;11(16):4808. doi: 10.3390/jcm11164808 (PMC9409718; doi:10.3390/jcm11164808)
Supplement: Supplementary file 1 [file jcm-11-04808-s001.zip › jcm-1811187-supplementary/Table S3.pdf]

Supplemental Table S3. Trend in general anesthesia use from 2005 to 2020 according to insurance and clinical definitions

| Fiscal year | N | Definition of general anesthesia, % of N (95%CI) | | |
|---|---|---|---|---|
| | | General anesthesia claim code (L008) (insurance definition of general anesthesia) | L008 with NMBAs (clinical definition of general anesthesia) | L008 without NMBAs |
| 2005 | 455 | 29.5 (25.3–33.6) | 9.9 (7.2–12.6) | 19.6 (15.9–23.2) |
| 2006 | 550 | 29.3 (25.5–33.1) | 7.5 (5.3–9.7) | 21.8 (18.4–25.3) |
| 2007 | 568 | 26.6 (23.0–30.2) | 8.6 (6.3–10.9) | 18.0 (14.8–21.1) |
| 2008 | 744 | 23.5 (20.5–26.6) | 5.1 (3.5–6.7) | 18.4 (15.6–21.2) |
| 2009 | 1109 | 21.3 (18.9–23.7) | 5.2 (3.9–6.5) | 16.1 (13.9–18.2) |
| 2010 | 1863 | 22.5 (20.6–24.4) | 4.7 (3.8–5.7) | 17.8 (16.0–19.5) |
| 2011 | 2567 | 23.8 (22.1–25.4) | 5.9 (5.0–6.8) | 17.8 (16.4–19.3) |
| 2012 | 3494 | 21.8 (20.4–23.1) | 4.4 (3.7–5.1) | 17.3 (16.1–18.6) |
| 2013 | 5274 | 16.7 (15.7–17.7) | 4.3 (3.8–4.9) | 12.4 (11.5–13.3) |
| 2014 | 5707 | 13.9 (13.0–14.8) | 4.4 (3.9–5.0) | 9.5 (8.7–10.2) |
| 2015 | 7264 | 12.9 (12.2–13.7) | 3.6 (3.2–4.0) | 9.3 (8.7–10.0) |
| 2016 | 8955 | 12.4 (11.8–13.1) | 3.5 (3.2–3.9) | 8.9 (8.3–9.5) |
| 2017 | 10511 | 12.2 (11.6–12.9) | 3.2 (2.8–3.5) | 9.1 (8.5–9.6) |
| 2018 | 12527 | 12.5 (11.9–13.0) | 3.7 (3.4–4.0) | 8.8 (8.3–9.3) |
| 2019 | 12109 | 12.1 (11.5–12.7) | 3.6 (3.2–3.9) | 8.6 (8.1–9.1) |
| 2020 | 2483 | 11.5 (10.2–12.7) | 3.3 (2.6–4.1) | 8.1 (7.1–9.2) |
| total | 76180 | 14.4 (14.2–14.7) | 3.9 (3.8–4.1) | 10.5 (10.3–10.7) |

CI, confidence interval; NMBAs, neuromuscular blocking agents
